# Dr. Clapp’s Rejoinder

**Published:** 1900-12

**Authors:** Dwight M. Clapp

**Affiliations:** 130 Commonwealth Avenue, Boston, Mass.


### Domestic Correspondence.

### DR. CLAPP'S REJOINDER.

To the Editor:

Sir,—Being a member of a State Board of Dental Examiners, I cannot let your editorial, "The Present and Future of State Boards," in the September number of your journal, go unchallenged. So far as my own board is concerned, your statements are in many particulars wrong and your conclusions unjust.

ABSTRACTS FROM EDITORIAL.

"It may seem to some impolitic to reopen a controversy with the organizations known by the name of State Boards of Dental Examiners, and this certainly would be unwise had there ever been a permanent peace established between the colleges and these political overseeing bodies. This, however, has never been the case; the quiet of the hour is that false quiet that precedes a more violent volcanic eruption.

"The Boards have all had their usual spring examinations, and a certain per

DR. CLAPP'S REPLY.

The Massachusetts Board when first appointed, in 1887, was one of those "political overseeing bodies" referred to.

Of the original appointees, two are still on the board and one died in office after having served for ten years. The other two members resigned.

After an association with three of these gentlemen in board examinations for years, and an acquaintance with the two resigned members for more than twenty years, I can truthfully and cent. of applicants have been rejected after having passed the various examinations of the college faculties.

"To the average laymen this means a wholesome protection to the public and a renewed assurance of the value of this supervisory care in preventing unqualified men from entering the professional ranks. To the educators behind the examined students, it means simply that these students have succeeded or failed to answer a sufficient number of ten or a dozen questions in each branch. The success does not secure the dear public from the imposition of inefficiency, nor does it prove that the rejected are incapable of rendering good practical service."

cheerfully say that they are as careful, capable, and conscientious examiners as are the average professors in dental colleges and schools. I base this statement on a personal acquaintance with a large number of dental professors.

I do not claim that our board is infallible, neither will I allow that examinations by dental college professors are infallible, but I contend that our board is a supplement to the colleges, and as it is in harmony with the colleges is an additional safeguard to the public.

"The reason for this is obvious to the educator, but may not be equally clear to the man who looks solely upon the surface of things. The list of ten questions before alluded to may cover each of the subjects of anatomy, physiology, chemistry, materia medica, pathology, therapeutics, bacteriology, metallurgy, histology, oral surgery, mechanical and operative dentistry. It is equally clear that if a man should fail before the State board on anatomy and physiology, materia medica, or chemistry, it would so reduce his average that he would be rejected, although his standing in the other branches, especially the practical, might place him in the front rank in those qualifications that would enable him to render good service to suffering humanity. This does not seem to be considered by the examiners in their final decisions, and the young man is cast out, having spent his three years and earned his diploma in vain. The responsibility of thus wrecking a life may be appreciated by some of the boards of examiners, but it is feared there is a certain degree of satisfaction felt by others that they can

Our board never rejected a man who failed "on physiology, materia medica, or chemistry" provided he was well up on the other and more practical branches, but it does reject men who show by their examination papers that they know little or nothing on all of these subjects, even if dental colleges have accepted their money and soft-hearted professors voted them diplomas, thereby informing a trusting public that they are competent to practise dentistry in their (the professors') estimation.

Our board believes that the public should receive as great consideration as a student, and is not in the gloating business over wrecked lives.

thus show their great superiority to the colleges that trained these young men. . . ."

"The questions usually asked bear only a remote relation to the several subjects as practically taught. This could not be avoided when the source of most of these is understood. Quiz compends and ancient first editions of Harris's "Principles and Practice" are not exactly the best mines out of which to dig thoughts and modern science. It is not to be understood that this is applicable to all members of examining boards. Many of these are known to be cultured men, anxious to do the right thing in the right way. These, however, are handicapped by their lack of knowledge of what a modern dental college education means. They, as a rule, have never held close relation with professional pedagogical work, and therefore are, and have always been, out of touch with that knowledge which can only be received through intimate relations with student life and students' work. The questions given in these re-examinations lack life. They are apt to be general in character, striking, as it were, at the periphery of a subject rather than seeking the innermost central idea. The untrained examiner invariably seeks a question that when answered means nothing of real value, and which fails to determine the knowledge of the individual, but it gives a certain degree of satisfaction to the examiner, as it magnifies his self-consciousness of power."

The following are the questions asked at our examination in June, 1900. They can be pulled to pieces, and are not above technical criticism. We never ask catch questions, our aim being to so word them that they may be easily comprehended by, and not confuse, the ordinary candidate.

I have been connected with a dental school for many years and am familiar with school examinations, and I submit these questions without hesitation, believing that they average in fairness and intelligence with any college questions on the same subjects.

OPERATIVE DENTISTRY.

(*June 20, 1900.*)

1. State briefly the general instructions you would give a patient in the hygienic care of the teeth.
2. What are the different shades of color in dental decay indicative of?
3. Describe in detail your method of destroying the pulp and your after treatment of the tooth.
4. What causes the discoloration of pulpless teeth? How would you avoid it?
5. State how you would distinguish between a case of pericementitis and acute alveolar abscess.
6. Give treatment of each case.
7. What conditions invite and are best served by combination filling? Describe process.
8. Describe your treatment of "pyorrhea" in its incipient and chronic stage.
9. When a filled tooth is particularly sensitive to heat, what does it indicate?
10. Describe the qualities that gold possesses to make it so desirable as a filling-material.

CHEMISTRY AND METALLURGY.

(*Examination, June, 1900.*)

1. Define (*a*) Cohesion, (*b*) Adhesion, (*c*) Polarity, (*d*) Repulsion.
2. What are the three laws of chemical combination?
3. Where does Nitrogen occur? What are its properties? Why does Nitrogen often produce explosive compounds?
4. Finish the following equation: $CaCo_3 + H_2SO_4 =$ . Describe the process represented. What are the properties of the gas thus formed?
5. What is Borax? Where does it occur? What are its properties?
6. What effect on Gold to alloy it with (*a*) Tin, (*b*) Lead, (*c*) Bismuth, (*d*) Silver?
7. How is Copper Amalgam prepared? What serious objections against its use for fillings in the mouth?
8. How is *elastic* or *spring* gold prepared, and for what purpose is such an alloy used in dental practice?
9. State the advantages and disadvantages of an alloy of Platinum and Gold for filling teeth.
10. What is Purple of Cassius? How is it prepared? Its use in dentistry?

MATERIA MEDICA AND THERAPEUTICS.

(*Examination, June, 1900.*)

1. What is an Aqua? A Liquor? A Spiritus? A Tinctura? An Extractum Fluidum? Give an example of each.
2. What is a Diaphoretic? A Diuretic? An Antiphlogistic? A Sialogogue? An Anodyne?
3. What is the action of Acids and Alkalies on the secreting glands?

4. Mention two heart stimulants that act rapidly and give the dose of each.
5. Describe Chloral; physiological action and dose; its effect on the blood; what effect when used hypodermically in the gums?
6. Describe the therapeutics of astringents.
7. Describe the action of Iodoform in the treatment of putrescent canals and alveolar abscess. Name two substitutes for Iodoform.
8. Describe Belladonna; its physiological action; dose of extract and of tincture. Name the officinal alkaloid. What drug does it antagonize? Of what value as an anodyne application to an inflamed or exposed pulp?
9. Describe Ergot; physiological action; state when and for what purpose it might be used in dental practice. What preparation and dose?
10. Write a prescription for fifteen powders, containing Antipyrinum, Phenacetinum, Quininæ, Sulphas, Zingiber (pulv.), Caffeina Citrata. One powder to be given every two hours. The dose of Caffein to be one grain, and of the other drugs two grains each. State when and for what purpose this would be given in dental practice.

PHYSIOLOGY AND ANÆSTHESIA.

(*June 20, 1900.*)

1. Describe reflex odontalgia.
2. What is the function of the heart Liver? Kidney? Pancrea?

3. Describe the formation of the enamel organ?
4. What is the origin of the uric acid in the body; conditions that increase its production?
5. Describe the structure of a salivary gland.
6. What are the organisms that produce pus? What is pus?
7. Name the digestive fluids and state their function.
8. How is nitrous oxide made and prepared for administration?
9. State the general instructions you would give a patient to prepare for the administration of ether.
10. What are the signs of danger in ether narcosis?

PROSTHETIC DENTISTRY.

(*June 20, 1900.*)

1. Mention three materials for artificial plates, giving special conditions for which each is indicated.
2. Write one-half page on diagnosis and prognosis of a mouth for which an artificial denture is called for.
3. *a.* Explain what "suction" is as spoken of in connection with artificial dentures.

   *b.* Give some of the difficulties in the way of obtaining a good suction.
4. Describe (*a*) Hammer swedging.
   (*b*) Press swedging.
   (*c*) Shot swedging, and comment on each.
5. Give a short description of gold plate, platinized gold and gold solder used in prosthetic dentistry.
6. What is a cast metal plate, and how is it made?
7. What are the principal objections to the soldered gold dentures formerly so extensively used, and how may these objections be overcome?

8. How would you restore the inferior molars and second bicuspids for a man with a normal jaw?
9. In flasking a vulcanite case state under what conditions you would have the teeth remain in the half of the flask with the model, and when you would have them come away in the other half.
10. If in swedging a gold plate some of the metal of the die should adhere to the gold, or, if a piece of tin or a globule of mercury should become attached to the plate, what would be the result when the gold is again annealed?

PATHOLOGY, SURGERY AND BACTERIOLOGY.

(*June 20, 1900.*)

1. Mention the varieties of inflammation of the mucous membrane. Give the appearance of *one* variety.
2. Give the treatment of contaminated wounds.
3. What are the local and constitutional causes of secondary hemorrhage?
4. Describe the pathologic changes in acute inflammation.
5. Describe the changes produced by luxation of the temporo-maxillary articulation.
6. What are the causes, symptoms and treatment of periostitis of the lower jaw?
7. Describe tracheotomy. When is the operation indicated?
8. What are the different classes of bacteria? Describe them.

9. Mention the different kinds of tumors of the gums.
10. Why should dental instruments be sterilized? Give your method.

ORTHODONTIA—CROWN AND BRIDGE WORK.

(*June 20, 1900.*)

1. *a.* How would you elongate a tooth?
   *b.* How would you depress a tooth?
2. *a.* How would you rotate a tooth?
   *b.* How hold it in position after rotating?
3. How would you make a crib plate? Give some of its uses.
4. Name and describe (not how made) all the crowns you can.
5. How would you crown a superior cuspid root for a lady, the root being strong and healthy?
6. How would you crown an inferior second bicuspid root for a man, the root being strong and healthy?
7. It being imperative to crown a superior central root, the root having a small hole through its side midway between its apex and the margin of the gum, how would you do it?
8. What is the difference between a removable bridge and a plate with clasps?
9. How would you bridge in the six superior anterior teeth, having the left cuspid right central and lateral roots, all the roots being firm and healthy, *but out of their normal positions?*
10. If you were making bridges having for their abutments the inferior left cuspid and second molar, and superior left cuspid and second molar (both in same mouth), how would you get the articulation?

ANATOMY AND HISTOLOGY.

(*June 20, 1900.*)

1. What blood vessels supply roof of the mouth, where do they emerge, and where do they make their exit?
2. What bones form naso-lachrymal canal, and where does it open?
3. What does infra orbital artery anostomose with?
4. Give course of facial artery and its branches.
5. Give function of buccinator muscle, with its origin and insertion.
6. Locate, describe and give distinguishing features of Femur and Ulna.
7. Nerves—point of exit from cranium, character of and general distribution of fifth and ninth pairs.
8. Describe the structure (*a*) of bone; (*b*) of dentine; (*c*) of enamel.
9. Describe the varieties of muscular tissue.
10. Describe the odontoblasts.

"The question of greater importance to the public, were it properly appreciated, would be, Do these re-examinations protect the people? As the boards are at present constituted, the answer must be in the negative. They not only do not protect, but experience has demonstrated that they are a menace to the very people they are supposed to serve."

The experience of our board is that these "re-examinations" are absolutely necessary. How it would be if all professors were like our editor and all schools like his I do not pretend to say, but as the *schools* "are at present constituted" it is the only way.

We have scores of men come up for examination holding diplomas from various colleges, who, if their work before us is any test, do not know the first principles of cavity formation or of gold manipulation; they cannot diagnose the plainest case of a dead pulp, but fill root-canals in the upper jaw (one recommended this) by turning the patient's head upside down so that the filling-material might flow into the canal by gravitation. Yet these men hold the degree of D.D.S. voted to them by professors in dental colleges.

These are the men our board keeps from practising dentistry in this State by re-examination. In what sense can this be "a menace to the very people they (the boards) are supposed to serve?"

"A quarter of a century has passed since the influence of State boards began to be seriously felt, and this constitutes a sufficient period to measure results. Twenty-five years ago there were a few cases in all large cities where men sought to secure practice by unethical methods, but these were so few, and, while annoying from a professional stand-point, they occasioned no marked deleterious influence. The result now, after this long period of law, is that the "Dental Parlor" is duplicated and reduplicated in every large city, and even in smaller towns where it previously had no existence. It may be said that this has no direct bearing on the laws; that this would have been the result had no laws governing dentistry been placed upon the statute-books. This is possibly true, but the laws have been a complete failure in the suppression of these, even in the one State that fully enforces its law. The graduates, instead of being fewer in number, have been enormously increased until even educators have become anxious as to the final result. To meet this and to make more thorough men, the standard of entrance has been raised in all the colleges of the country. The curriculum has been advanced and the time lengthened until it would seem impossible to go farther in this direction, and yet a still further increase in years is now demanded. For all these changes the boards take to themselves special credit. It is safe to assert that they had nothing to do with any ad-

Our editor has in this paragraph completely demolished, in his own estimation, all State boards. He, at least, has no further use for them.

Colleges will continue to graduate quacks just so long as they will pay their money, behave themselves while in college, and pass the required examination.

The larger number of quacks, however, probably never saw the inside of a college. That the public will be less injured by quacks that can pass a good stiff board examination than by those who cannot is hardly open to question. For this class we cannot provide too many examinations.

If all this "advance" has "come from within," why do the professors of dental colleges come to us and ask what they can do to fit their students to pass our boards? Why do they wish to know wherein their students are deficient when they come before us for re-examination? Our editorial says that no influence on the standard of the colleges comes from "without." The boards are certainly not "within," yet we are asked to advise in the matter of the college standard.

What has caused this "enormously increased" number of graduates that makes "even educators . . . anxious?" Members of examining boards *know* that a large number of men go to the dental colleges for the sole purpose of enabling them to pass State board examinations. This is *beyond question* one great cause of the increase in the numvance. This came from within, and not from without, and would have been found necessary had the boards never been established. This is the status at present. The students are made to suffer, and the dental profession has gained nothing through this so-called supervisory power."

ber of students. Now if dental education is a good thing and tends to make better and more competent dentists, the public gains through the existence of State boards that which the college training represents for all the men who are influenced by them to attend college.

Does our editor believe that this extra amount of dental college training is a safeguard or a menace to the public?

In the last line of this paragraph the fact that "the dental profession has gained nothing" is bemoaned. In this State the law was not passed for the benefit of the profession, and it is not being administered on that line.

"The fact that the State laws governing dentistry are not enforced to any appreciable extent is patent to every observer. It is possible in some of the States possessing stringent laws, for a man to practise in spite of the boards, and this has frequently been done. The answer the boards make to this is, "We are not appointed for police work, and fulfil our duty when we have examined the students." This is true, and the enforcement of the law then devolves on others. The State officials whose duty it is take no interest in it, and will do nothing unless cases are brought directly to their notice, with proper evidence sufficient to secure conviction. The State societies have appointed committees, but they have failed to produce satisfactory results. The duty is disagreeable, and means large expenditure of money and time, and a certain loss of reputation. The result is that the law is not enforced. It would be perfectly easy in the State in which the writer lives for a man to practise dentistry without even a college training, and some have boldly declared they would practise in spite of the decision of the board. This is unquestionably a bad state of things,

In Massachusetts there have been one hundred and forty-nine prosecutions. With three or four exceptions those prosecuted have each paid fifty dollars, or upward, fine.

A very large number who were practising illegally have stopped on being notified that they would be prosecuted if they continued to violate the law.

I invite our editor and all other men to assist in the enforcement of good dental laws in the spirit and for the purposes for which they were enacted.

There are in this State laws against theft, arson, embezzlement, pollution of water supplies, bigamy, and sundry other evils, and every day these are being broken, yet our good citizens do not say, "Better no law than its constant violation."

but it is not only the case, but there seems no prospect of any improvement. Better no law than its constant violation. . . ."

"The future of these boards is a problem that will require the combined wisdom of all thinking minds. It is too late to expect these to be dispensed with. The laws will remain, and we must continue to have these bodies to deal with for many years to come. The question that interests us now is, Can there be any change made in the selection of those who will constitute these boards in the future?"

In this State there are two dental schools. The relations of the board to these schools is most gratifying, each being respected by the other, and all working in harmony.

It sometimes happens that the board turns down a man that one of the schools has graduated. An investigation almost always shows that the man was just on the border-line, and it was a question for discussion whether he should receive a degree or not. So, also, if we pass a man to whom the school has refused its diploma, we find that he went in by the smallest margin, thus showing that the estimate of the schools and that of the board were almost exactly on the same line.

There is one notable instance where a diploma was granted by one of the schools and a certificate of registration by the board to a man who was very deficient in certain qualifications usually required but waived in this case on account of the almost universal belief of professors and examiners that he was an eminently *safe* man.

What is needed, as it seems to me, is that the same harmony of purpose and mutual regard that exists between schools and board in this State shall be brought about between all schools and all boards in all of the States.

This can be done only when all good men and especially such strong men, of which our editor is the type, shall drop prejudice and not close their eyes to existing good, but, with true loyalty to the people, to the professson, and to the student, work in harmony for the perpetuation of that which is good in present laws and the pruning out of the bad

I am acquainted with many dental educators, and I know that they are trying to do their best. I am acquainted, also, with dental examiners in several of the States, and from their standing as professional men and as gentlemen I know that they are just as zealous, faithful and honest in the discharge of their duties as are the professors.

Let us foster in every way the better understanding that is gradually growing between the National Association of Dental Faculties and the National Association of Dental Examining Boards.

Does any one think that without State boards the number of those calling themselves dentists would not be largely increased? Six months in a dentist's laboratory would be considered by many ample preparation for opening an office and prefixing the title of Dr. to their names. To this class State boards prove a wholesome stumbling-block, and I am sure the people of this State would not consent to have the law repealed.

DWIGHT M. CLAPP, D.M.D.

130 COMMONWEALTH AVENUE, BOSTON, MASS.

